# Histone H3 Acetylation at *Sox1ot* Promoter by Targeted Epigenome Editing Augments Proliferation of Intermediate Progenitors in Developing Cortex

**DOI:** 10.3390/biology15141110

**Published:** 2026-07-09

**Authors:** Godwin Sokpor, Pauline Antonie Ulmke, Hoang Duy Nguyen, Linh Pham, Princy Kakani, Van Trung Chu, Sebastian J. Arnold, Beate Brand-Saberi, Huu Phuc Nguyen, Tran Tuoc

**Affiliations:** 1 Department of Human Genetics, Ruhr University of Bochum, 44791 Bochum, Germany; pauline.ulmke@ruhr-uni-bochum.de (P.A.U.); hoang.nguyen-j79@ruhr-uni-bochum.de (H.D.N.); linh.pham@ruhr-uni-bochum.de (L.P.); princy.kakani@ruhr-uni-bochum.de (P.K.); huu.nguyen-r7w@ruhr-uni-bochum.de (H.P.N.); 2 Lincoln Medical School, University of Lincoln, Lincoln LN6 7TS, UK; gsokpor@lincoln.ac.uk; 3 Max Delbrück Center for Molecular Medicine in the Helmholtz Association, 13125 Berlin, Germany; vantrung.chu@mdc-berlin.de; 4 Institute of Experimental and Clinical Pharmacology and Toxicology, Faculty of Medicine, University of Freiburg, 79104 Freiburg, Germany; sebastian.arnold@pharmakol.uni-freiburg.de; 5 CIBSS—Centre for Integrative Biological Signaling Studies, University of Freiburg, 79104 Freiburg, Germany; 6 Department of Anatomy and Molecular Embryology, Institute of Anatomy, Medical Faculty, Ruhr University Bochum, 44801 Bochum, Germany; beate.brand-saberi@rub.de

**Keywords:** H3K9 acetylation, intermediate progenitors, lncRNA *Sox1ot*, epigenome editing, CRISPR-dCas9, brain development

## Abstract

Factors regulating the genesis and expansion of basal progenitor cell sub-populations, including intermediate progenitor cells, are critical determinants of cortical neurogenesis and brain growth. Epigenetic (chromatin) marks have emerged as notable regulators of cortical development. However, it is unclear how these factors specifically interact in the epigenome to orchestrate brain development. Here, we combined in vivo electroporation and a CRISPR-dead (d)Cas9 system to probe the contribution of a specific histone modification mark, H3K9ac (H3K9 acetylation), in the epigenome of isolated intermediate progenitor cells in developing mouse cortices. CRISPR-dCas9-mediated addition of H3K9ac at the promoter region of lncRNA *Sox1ot* resulted in *Sox1ot* upregulation, with attendant increase in the intermediate progenitor pool and augmented neurogenesis. Thus, we found an interplay between H3K9ac and *Sox1ot*, which drives the amplification of cortical intermediate progenitor cell population. By targeting H3K9ac to the *Sox1ot* promoter, we were able to stimulate the proliferation of intermediate progenitor cells leading to enhanced cortical neurogenesis. In essence, we have identified H3K9ac as a key epigenetic regulator of *Sox1ot* expression, which may dynamically be involved in intermediate progenitor cell pool expansion during brain development and evolution.

## Simple Summary

The abundance of a sub-group of originative cortical neural cells called basal progenitors is critical for the production and expansion of the neuronal population during brain development. However, our understanding of how basal progenitor cell proliferation is epigenetically regulated is largely unclear. We set out to determine the involvement of histone acetylation and *Sox1ot*—a long non-coding RNA—in the epigenetic regulation of cerebral cortex development. To achieve this, we established an epigenome-editing protocol that allowed us to install a specific histone acetylation mark (H3K9ac) at the promoter region (a regulatory domain) of the *Sox1ot* gene exclusively in a sub-type of basal progenitors called intermediate progenitor cells in the developing mouse cortex. Such experimental enrichment of H3K9ac at the *Sox1ot* promoter augmented *Sox1ot* gene expression, leading to increased proliferation of intermediate progenitors and enhanced cortical neurogenesis. Thus, we identified a downstream effect of H3K9ac mediated by *Sox1ot* in driving the amplification of intermediate progenitor cells during cortical development. The findings unravel a critical regulatory axis which underpins cortical growth and hence deepens our understanding of the epigenetic modulation of brain development.

## 1. Introduction

The epigenome presents an additional layer of gene regulation without genomic sequence alteration, and it is capable of influencing cellular phenotypes in a stable and inheritable manner [1]. A prominent feature in the epigenome is the activity of histone-modifying enzymes, which can dynamically remodel the epigenetic landscape in response to inherent cellular demands and/or environmental stimuli. Such chromatin modifying factors have enzymatic functions with which they can either install or erase chromatin-associated elements to regulate chromatin accessibility and therefore modulate gene expression [2].

The acetylation (addition of acetyl group) or deacetylation (removal of acetyl group) of histones by the enzymes acetyltransferases and deacetylases, respectively, are well-known chromatin modifying mechanisms which sculpt the distribution of acetyl histone or chromatin marks along gene bodies [3]. The localization of acetyl chromatin marks, especially at regulatory regions of genes (promoter, enhancers), can modulate gene expression to elicit pertinent cell phenotypes [4].

One strategy for exploring the biological significance of the dynamic installation of chromatin marks in the epigenome is to rationally control their distribution and study the resulting effect. To that end, many techniques have been developed to manipulate chromatin-related factors, including traditional knockdown, knockout, and overexpression methods. However, these approaches have limitations in terms of efficiency, specificity, and activity. The growing effort to improve such experimental strategies has led to the development of epigenome editing platforms with improved outcomes in manipulating single or multiple chromatin marks without disturbing the nucleotide sequences in the genome. In general, epigenome editing tools employ programable DNA-targeting modules which can bring coupled effectors (epiEffectors) to gene loci to carry out desired epigenetic modifications [5].

In this study, we applied the CRISPR-Cas9 (Clustered regularly interspaced short palindromic repeats [CRISPR], and CRISPR-associated sequences [Cas9]) platform to effect epigenome editing of histone acetylation at a target gene locus. To conduct cell type-specific epigenome editing, we referred to our recent report that H3 acetylation in mammalian basal progenitors is a notable mechanism underlining the evolutionary complexification of the neocortex [6]. Thus, we were guided by this finding to perform experimental manipulation of H3 acetylation in the acetylome of intermediate progenitor cells (IPCs) in the developing mouse cortex using the epigenome editing method.

We focused on the candidate gene *Sox1ot (Gm5607),* an lncRNA associated with the neurogenic transcription factor Sox1 and hereby referred to as *lncSox1* in this study, for our epigenome editing method because it was highlighted to be differentially expressed in TBR2−and TBR2+ cells following brain-wide treatment with the histone deacetylation chemical inhibitor Trichostatin A (TSA) to effect global H3 acetylation upregulation. To test if locally increasing histone acetylation in the promoter region of *lncSox1* could augment its expression, we designed an epigenome editing construct by including an *lncSox1* small-guidance (sg) RNA (*sglncSox1*), and a histone acetyltransferase KAT2A module which specifically targets histone 3 (H3) at the lysine position 9 (K9) for acetylation (i.e., H3K9ac). Upon co-transfection with a Cre recombinase encoding plasmid, the designed *gLncSox1-dCas9-KAT2A-T2A-eGFP* DNA fragment is fully transcribed leading to the assembly of a functional H3K9ac epigenome editing complex. With the resultant epigenome editing platform, we were able to efficiently add the chromatin mark H3K9ac to the promoter region of *lncSox1*, which resulted in increased expression of *lncSox1* in cultured Neuro2A cells. It was reasoned that in utero electroporation (IUE)-mediated transfection of IPCs with the H3K9ac-enriching *lncSox1* construct can allow for observation of the (neuro)biological phenotypic effect of H3K9ac-dependent *lncSox1* upregulation in the developing mouse cortex.

## 2. Materials and Methods

### 2.1. Transgenic Mice and in Utero Electroporation (IUE)

Tbr2/EomesCreER mice [7] were maintained in a C57BL6/J background. Animals were handled according to the German Animal Protection Law. The animal study protocols were approved by the research ethics committee of Bezirksregierung Braunschweig (14/1636, 16/2330, 18/3038, and 22/8102).

In utero electroporation was done as previously described [6,8,9,10] with modifications. Timed-pregnant Tbr2/EomesCreER mice were pre-treated with tamoxifen and used for electroporation at the indicated embryonic stages. Pregnant female mice were anesthetized by intraperitoneal injection of a ketamine/xylazine/acepromazine mixture. The abdominal area was sterilized with 70% ethanol, and a midline laparotomy was performed to expose the uterine horns. Plasmid DNA was diluted to 5 μg/μL in sterile PBS containing 0.01% Fast Green to visualize successful injection. DNA solution was microinjected into the lateral ventricle of embryonic brains using pulled glass capillaries connected to a mouth pipette. Approximately 1–2 μL of DNA solution was injected per embryo. Following injection, platinum tweezer electrodes (BTX, Harvard Apparatus, Holliston, MA, USA) were positioned around the embryonic head, and electric pulses were delivered using an ECM830 electroporator (BTX, Harvard Apparatus). For all electroporation experiments, five square-wave pulses of 50 ms duration with 950 ms intervals were applied. Voltage settings were adjusted according to embryonic stage: 30–31 V for E12.5–E13.5 embryos, 32–35 V for E14.5 embryos, and 35 V for E15.5 embryos. To minimize embryonic damage, only every second embryo within each uterine horn was electroporated, with a maximum of four embryos electroporated per pregnant dam. Throughout the procedure, exposed uterine horns were continuously moistened with pre-warmed sterile 0.9% NaCl solution. Following electroporation, the uterine horns were returned to the abdominal cavity, and the muscle and skin layers were separately closed using 5–10 absorbable sutures (Ethicon, Somerville, NJ, USA). Animals were allowed to recover on a 37 °C warming pad until fully awake before being returned to their home cages. Under these conditions, the procedure routinely achieved a transfection efficiency exceeding 30% within the targeted cortical region and an embryo survival rate above 70%. Transfection efficiency was determined by quantifying GFP-positive cells within the electroporated area, while embryo survival was assessed as the percentage of viable embryos recovered at the experimental endpoint relative to the number of embryos initially electroporated.

### 2.2. Plasmids

Plasmids used in this study: pCIG2-Cre-ires-eGFP (a gift from Prof Francois Guillemot, NIMR London), *sgLncSox1#1#2-LoxP-mCherry-PolyA-LoxP-dCas9-KAT2A-T2A-eGFP* (original to this study).

### 2.3. Antibodies

Monoclonal (mAb) and polyclonal (pAb) primary antibodies were commercially sourced and listed as follows: GFP chick pAb (1:400; Abcam, Cambridge, UK), mCherry/RFP pAb (1:1000, Rockland Immunochemicals, Limerick, PA, USA), mCherry/RFP mAb (1:1000, Rockland Immunochemicals), NeuN mouse mAb (1:200, Chemicon, Temecula, CA, USA), HuCD mouse mAb (1:20; Invitrogen, Carlsbad, CA, USA), Pax6 rabbit pAb (1:200; Covance, Princeton, NJ, USA), Pax6 mouse mAb (1:100; Developmental Studies Hybridoma Bank, Iowa City, IA, USA), TBR2 rat 923 mAb (1:200; eBioscience, San Diego, CA, USA), TBR2 rabbit pAb (1:200; Abcam), H3K9ac pAb (ChIP-seq, Millipore, Billerica, MA, USA), H3K18ac pAb (ChIP-seq, Abcam), pHH3 mAb (1:50; Cell Signaling Technology, Danvers, MA, USA), BrdU rat pAb (1:100; Abcam), BrdU mouse mAb (1:40; CalTag Laboratories, Burlingame, CA, USA), Tuj mAb (1:200; Chemicon). Secondary antibodies from various species used are Alexa (488, 568, 594 and 647)-conjugated IgG (1:400; Molecular Probes, Molecular Probes, Eugene, OR, USA).

### 2.4. Mouse Treatment with HDAC Inhibitors (HDACi) [6]

Trichostatin A (TSA) solution was prepared at a concentration of 100 µg/mL by dissolving in vehicle (8% ethanol in 1×PBS). Pregnant mice from E12.5 dpc were treated twice daily with either 150 µL of 100 µg/mL TSA solution or vehicle via intraperitoneal injection and sacrificed at various stages of embryonic development.

### 2.5. TBR2-Stained Nuclei and Cell Sorting from Embryonic Mouse Cortex [6]

#### 2.5.1. Sorting TBR2+ Nuclei for ChIP-Seq

The protocol was adopted from Halder and colleagues [11]. Unless stated otherwise, steps were done on ice or at 4 °C. Replicates of CD1 embryonic cortices (pooled from 5 pubs) were briefly homogenized with plastic pestles in 1.5 mL tubes in low-sucrose buffer (320 mM Sucrose, 5 mM CaCl2, 5 mM MgAc2, 0.1 mM Ethylenediamine tetraacetic acid (EDTA), 10 mM HEPES pH 8, 0.1% Triton X-100, 1 mM DTT, supplemented with Roche protease inhibitor cocktail). Incubation with 1% formaldehyde for 10 min at room temperature was used for crosslinking. Excess formaldehyde in the solution was neutralized with 125 mM Glycine under same incubation condition for 5 min. The mixture was centrifuged at 2000× *g* for 3 min, and the nuclear pellet was resuspended in low-sucrose buffer and mechanically homogenized further with a homogenizer (IKA Ultraturax-Werke GmbH & Co. KG, Staufen, Germany). The mixture was sedimented in high-sucrose buffer (1000 mM Sucrose, 3 mM Magnesium acetate, 10 mM HEPES pH 8, 1 mM DTT, protease inhibitor) by spinning in Oak-Ridge tubes at 3200× *g* for 10 min in a swinging bucket rotor centrifuge. This effectively eliminates myelin. The supernatant was carefully decanted, and nuclei were collected in 2 mL DNA-low bind microfuge tubes following 3 min centrifugation at 2000× *g*. Excess sucrose was removed, and the pellet of nuclei resuspended with 500 uL PBTB buffer (PBS-0.2% Tween-1% BSA buffer, with protease inhibitor). Application of anti-TBR2 for 1 h was used to achieve Tbr2 staining followed by washing of nuclei and resuspension with 500 µL PBTB. Tbr2 nuclei sorting was performed using FACSAria III. Samples with no antibody were used as negative control for gating. Sorted nuclei were collected into 15 mL falcon tubes coated with PBTB and briefly centrifuged for the Tbr2 stained and unstained nuclei to pellet. Liquid nitrogen was used to flash-freeze the sorted nuclei pellet which was subsequently stored at −80 °C for later use in the ChIP experiment.

#### 2.5.2. Sorting TBR2+ Nuclei for RNA Isolation and Sequencing

Freshly dissected CD1 embryonic cortices (pooled from 5 pubs) were collected and immersed in RNAlater solution contained in a microfuge tube, which was kept at 4 °C for not less than 24 h. Two washes with 1× RNAse free PBS were enough to remove excess RNAlater solution from the cortices. Incubation steps were performed on ice and centrifugation at 500× *g* in 4 °C, where not specified. The cortical tissues were homogenized with 500 uL lysis buffer (NUC101, Sigma-Aldrich) using 30–45 plastic pestles agitations. The homogenate was made to 2 mL by adding lysis buffer. Incubation was done for 7 min, after which the lysate was centrifuged for 5 min and nuclei pellet resuspended in 2 mL lysis buffer. The lysate was incubated for 7 min, filtered into a new 2 mL tube using a 40 µm filter, and the supernatant removed by centrifugation. The resultant nuclei pellet was washed with 1800 ul nuclei suspension buffer (NSB), centrifuged, and resuspended in 500 uL NSB. The NSB was prepared as follows: 0.5% RNAse-free BSA (Millipore), 1:200 RNaseIN plus RNAse inhibitor (Promega, Madison, WI, USA), protease inhibitor diluted into RNAse free PBS (Invitrogen, Carlsbad, CA, USA). Nuclei were stained for Tbr2 by applying anti-TBR2 Alexa 488 conjugated antibody for an hour. Stained nuclei were NSB-washed and resuspended in NSB. Tbr2 stained/unstained nuclei were then sorted into NSB coated falcon tubes for ChIP-seq as described in the section above. For RNA isolation, Trizol LS solution was added to nuclei collected by brief centrifugation. This was followed by chloroform addition for nuclei solubilization, and 15 min centrifugation at 120,000× *g*. Zymo RNA clean & concentrator-5 kit then was used to purify RNA from the collected aqueous phase. By means of an RNA library kit (Takara SMART-Seq v4 Ultra Low Input RNA kit, Takara Bio Inc., Kusatsu, Shiga, Japan) mRNA-seq libraries were prepared using 1 ng of RNA purified from the isolated Tbr2-labelled nuclei. Sequencing of the libraries was done in an Illumina Hiseq 2000 machine (Illumina, San Diego, CA, USA).

### 2.6. Chromatin Immunoprecipitation (ChIP)

The ChIP experiment was performed on FACS-isolated TBR2+ basal progenitors or GFP+ cells as previously described with minor modifications [12]. Briefly, cells were homogenized in sucrose buffer (0.32 M sucrose, 5 mM CaCl_2_, 5 mM Mg(Ac)_2_, 0.1 mM EDTA, 50 mM HEPES pH 8.0, 1 mM DTT, and 0.1% Triton X-100), followed by fixation with formaldehyde and quenching with glycine. Nuclei were isolated by centrifugation, washed in Nelson buffer, and lysed in RIPA-SDS buffer. Chromatin was fragmented by sonication using a Bioruptor Plus NGS sonicator (Diagenode, Seraing, Belgium) operated at high power at 4 °C. Following centrifugation, the soluble chromatin fraction was collected and stored at −80 °C. Chromatin quality and fragmentation efficiency were assessed after reverse cross-linking using an Agilent 2100 Bioanalyzer (Agilent Technologies, Santa Clara, CA, USA), while DNA concentration was determined using a Qubit fluorometer (Thermo Fisher Scientific, Waltham, MA, USA). For immunoprecipitation, Protein A Dynabeads (Thermo Fisher Scientific, USA) were blocked with bovine serum albumin (BSA) and used to pre-clear diluted chromatin samples. Pre-cleared chromatin was incubated overnight at 4 °C with antibodies against H3K9ac (07-352, Millipore) or H3K18ac (ab1191, Abcam). For ChIP-seq experiments, 1000 ng chromatin and 2 μg antibodies were used per reaction, whereas ChIP-qPCR experiments were performed with 500 ng chromatin and 1 μg antibody. Immune complexes were captured with blocked Dynabeads, followed by sequential washes with immunoprecipitation and high-stringency wash buffers. DNA was then eluted, treated with RNase A and Proteinase K, and reverse cross-linked overnight at 65 °C. Following purification using SureClean reagent (Bioline, London, UK), DNA was recovered in elution buffer. Input samples were processed in parallel under identical conditions. The quantity and quality of immunoprecipitated DNA were determined using a Qubit fluorometer and Agilent 2100 Bioanalyzer prior to downstream ChIP-seq library preparation or quantitative PCR analysis.

Immunoprecipitated DNA was analyzed either by qPCR or sequencing, in which Illumina sequencing libraries were generated with the DNA library preparation kit NEBNext Ultra II. An Illumina Hiseq 2000 machine was then used to sequence the libraries.

### 2.7. ChIP and RNA Sequencing Data Analyses

Next generation sequencing and data analyses were performed according to steps described in our previous work [6]. RNA-seq was performed and analyzed using our routinely established protocol described in our previous work [12,13].

### 2.8. Identification of lnRNAs Enriched in IPCs

The IPC genes with significant expression changes (log2FoldChange > 1.0, *p*-value < 0.01) were analyzed with the MGI gene nomenclature tool available at http://www.informatics.jax.org/batch (accessed on 15 October 2022). A list of genes which encode for lnRNAs proteins was extracted from each other.

### 2.9. Histochemistry Validation

Immunohistochemistry (IHC) was performed as previously described [8,14,15]. Briefly, embryonic mouse brains were dissected in cold phosphate-buffered saline (PBS) and fixed overnight at 4 °C in 4% paraformaldehyde (PFA) prepared in PBS. Following fixation, tissues were cryoprotected in 30% sucrose in PBS at 4 °C until fully equilibrated, embedded in Tissue-Tek O.C.T. compound (Sakura Finetek, Torrance, CA, USA), and cryosectioned at 12–20 μm thickness using a cryostat (Leica Microsystems, Wetzlar, Germany). For immunohistochemical analysis, sections were washed in PBS and permeabilized with 0.3% Triton X-100 in PBS. After blocking in PBS containing 5% normal donkey serum and 0.1% Triton X-100 for 1 h at room temperature, sections were incubated overnight at 4 °C with primary antibodies diluted in blocking solution. The primary antibodies were used as indicated. For experiments involving triple immunofluorescence labeling of mCherry-expressing tissues, endogenous mCherry fluorescence was eliminated prior to antibody staining by treatment with hydrochloric acid (HCl), thereby preventing interference with the detection of other fluorescent signals. Sections were subsequently washed extensively with PBS before proceeding with the standard immunostaining protocol. After primary antibody incubation, sections were washed three times in PBS and incubated with species-appropriate Alexa Fluor-conjugated secondary antibodies (Thermo Fisher Scientific) for 1–2 h at room temperature. Nuclei were counterstained with DAPI (4′,6-diamidino-2-phenylindole), and sections were mounted using Fluoromount-G mounting medium (SouthernBiotech, Birmingham, AL, USA).

### 2.10. qRT-PCR Analyses

Western blot analyses and qRT-PCR were performed based on our previously described protocols [9]. Primers used are shown in Table S6 and the RT^2^ Profiler PCR Array profiles (Qiagen, Hilden, Germany).

### 2.11. Epigenome Editing

#### 2.11.1. Design Epigenome Editing Constructs

The backbone vector (U6-sgMCS-CAG-LoxP-mCherry-PolyA-LoxP-dCas9-KAT2A-T2A-eGFP) was commercially synthesized by GenScript. gRNA sequences targeting specific mouse *lncSox1* promoter region (chr8:12383591-12387729) were designed using the software ‘Genious’ and inserted into a T7 promoter-based vector.

#### 2.11.2. Testing for lncSox1 sgRNAs, Quantification of H3K9ac Level at lncSox1 Promoter and Expression of lncSox1 in sgLncSox1-CAG-LoxP-mCherry-PolyA-LoxP-dCas9-KAT2A-T2A-eGFP-Transfected Neuro2A

Synthesized sgRNAs were tested in vitro. The PCR-produced sgRNA template used for the sgRNA synthesis carried the T7 promoter added by the gRNA sequence and the sharped gRNA scaffold. The in vitro transcription was done using the transcription kit MEGAscript T7 and following the manufacture’s protocol. Cas9 protein was sourced from Integrated DNA Technologies, Coralville, IA, USA (#1074182). The cutting efficiency of Cas9/gRNAs complexes was tested in vitro using an IPC PCR product from *lncSox1* promoter. Detailed description of the protocol used for the in vitro testing is available in our previous work [6]. After in vitro test, the sgLncSox1(#1, GTGTCTCGAACTCGCGCGCGG) and sgLncSox1(#4, GTGTGTGCCGAACGAGGAGCA) sequences were selected, synthesized and cloned into the backbone vector (U6-sgMCS-CAG-LoxP-mCherry-PolyA-LoxP-dCas9-KAT2A-T2A-eGFP) to generate corresponding plasmids (*sgLncSox1#1, sgLncSox1#4*). The vectors were sequenced for confirmation. Neuro2A cells were transfected with 12 µg of parental vector pCAG-Cre-ires-eGFP, or/and *sgLncSox #1*, *sgLncSox* #4 using lipofectamine 2000 reagent (Thermofisher) and cultured on 10 cm dishes. The cultured cells were collected 3 days after transfection, and FACS analyzed for qPCR and ChIP/qPCR analyses.

### 2.12. Microscopy and Statistical Analysis

Histological images were obtained using confocal fluorescence microscopy (TCS SP5, Leica) and analyzed with Axio Imager M2 (Carl Zeiss, Oberkochen, Germany) with a Neurolucida system. Further processing of micrographs was done using Adobe Photoshop. Quantities were averaged from a minimum of three biological replicates. Statistical analyses for histological experiments are detailed in Table S5.

## 3. Results

### 3.1. Histone Deacetylase Inhibition Altered the Expression of Non-Coding RNAs in Cortical Intermediate Progenitors

**Figure 1:**
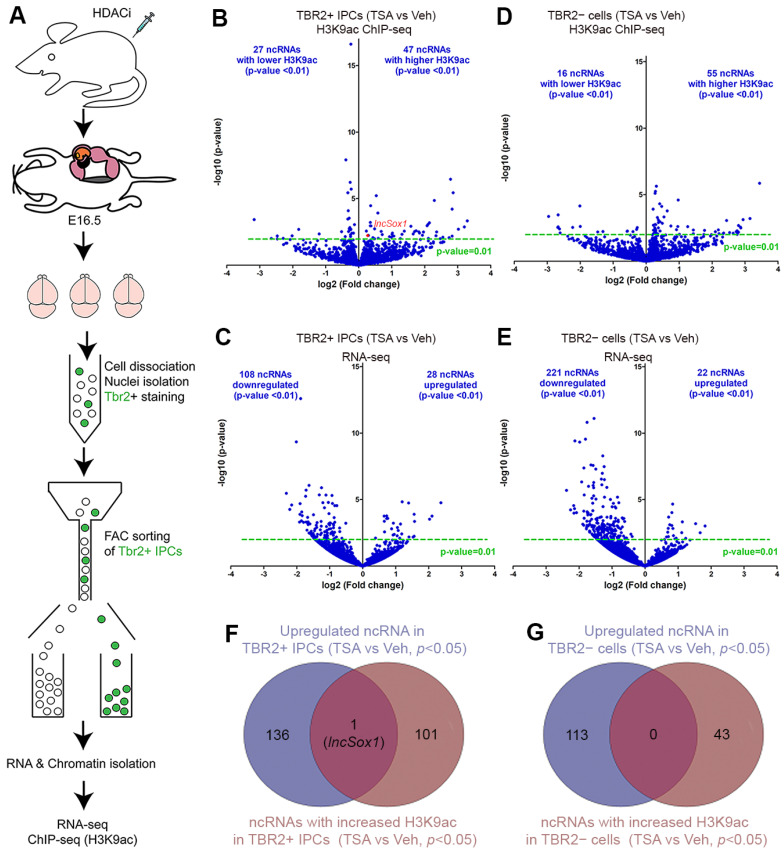
Alteration in the ncRNA milieu in IPCs due to global histone deacetylase inhibition. (**A**) Schematic overview of the protocol used for profiling the transcriptome and epigenome of IPCs after TSA treatment. Green and white circles represent TBR2-expressing (TBR2+) and non-TBR2-expressing (TBR2−) cells, respectively. (**B**–**E**) Volcano plots showing upregulated and downregulated ncRNA genes in terms of their association with the H3K9ac mark (**B**,**D**) and expression (**C**,**E**) after TSA treatment of IPCs (**B**,**C**) and non-IPCs (**D**,**E**) compared with their vehicle-treated counterparts. (**F**,**G**) Overlap between upregulated ncRNA genes and ncRNA genes with increased H3K9ac due to TSA-treatment of TBR2+ IPCs (**F**), and TBR2− IPCs (**G**). Abbreviation: IPCs, intermediate progenitor cells; TSA, Trichostatin A; HDACi, Histone deacetylation inhibitor.

Neocortex expansion is underscored by the enhanced proliferative potential of IPCs partly through a likely evolution-driven increase in H3K9 acetylation (H3K9ac) in mammalian IPCs [6]. To determine how H3 acetylation affects IPC biogenesis and/or proliferative capacity, we considered analyzing the IPC epigenome and transcriptome for downstream factors attributable to the prominent involvement of IPCs in cortical expansion. To achieve this, we globally increased H3 acetylation in the embryonic mouse brain by treatment with the selective class I/II histone deacetylase inhibitor (HDACi) Trichostatin A (TSA) via a daily intraperitoneal injection of pregnant wild-type mouse from E12.5 to E16.5 (Figure 1A) [6].

The TSA-treated E16.5 embryonic brains were harvested and processed for IPC isolation via a FACS protocol based on intranuclear immunostaining of IPCs [11]. We used the TBR2 antibody to label the IPCs in single-cell suspensions and subsequently isolated the TBR2 positive (TBR2+) cells from non-IPCs [i.e., TBR2 negative (TBR2−) cells] (Figure 1A) [11,16]. For comparative analysis, the sorted cells were matched such that TBR2+ and TBR2− cells treated with TSA were compared with TBR2+ and TBR2− cells treated with vehicle (Veh), respectively.

To determine how H3K9ac drives IPC generation in the developing cortex, we probed for epigenomic and transcriptomic signatures in IPCs against non-IPCs with or without TSA treatment. Thus, we performed chromatin immunoprecipitation sequencing (ChIP-seq) and RNA sequencing (RNA-seq) to profile the genome-wide distribution of H3K9ac and H3K9ac-dependent gene expression in the sorted cells. We observed that TSA treatment of the developing brain resulted in notable changes in the expression of both protein-encoding genes [6] and also genes encoding for ncRNAs in both treated IPCs and non-IPCs (Figure 1B–E).

Notably, the TSA treatment of TBR2+ IPCs evoked upregulation of H3K9ac partly related to the loci of 47 ncRNAs as opposed to 27 ncRNAs which displayed lower H3K9ac distribution (*p*-value < 0.01) (Figure 1B, Table S1). At the transcriptome level, we found that 28 ncRNA transcripts are upregulated, whereas 108 ncRNA transcripts are downregulated upon inhibition of H3 deacetylation in IPCs (*p*-value < 0.01) (Figure 1C and Figure S1, and Table S2). On the other hand, 55 and 16 ncRNAs in TBR2− cells displayed high and low H3K9ac, respectively (*p* < 0.01) (Figure 1D, Table S3). However, our gene expression analysis revealed that 22 ncRNAs are upregulated and 221 ncRNAs are downregulated in such non-IPCs without TSA treatment (*p*-value < 0.01) (Figure 1E, Table S4). Indeed, some ncRNAs are prominently expressed in non-Tbr2 expressing cells in the presumptive cortical plate (Figure S1).

To identify potentially direct ncRNA targets of H3K9ac, we compared the upregulated ncRNAs (*p*-value < 0.05) and ncRNAs with increased H3K9ac level (*p*-value < 0.05) in response to TSA treatment in TBR2+ IPCs (Figure 1F) and in TBR2− cells (Figure 1G). Interestingly, our comparison revealed lncSox1 as a potential downstream factor of H3 acetylation ascribable to the amplification of IPCs in the developing brain (Figure 1F). Together, our observations here show that H3K9ac enrichment in IPCs leads to alterations in the ncRNA landscape therein, with an impressive observation that the ncRNA *IncSox1* may be directly targeted for upregulation in IPCs following global inhibition of H3 deacetylation in the developing mouse brain.

### 3.2. TSA Treatment Increased Promoter H3K9ac Level and Expression of lncSox1 in IPCs

Following a fine screening of the ncRNA output of our ChIP-seq and RNA-seq analyses, we found *lncSox1* to be the only ncRNA with a significantly (*p* < 0.05) high H3K9ac and gene expression upregulation in IPCs due to TSA treatment (Figure 1F). However, in the case of non-IPCs treated with TSA, there was no specific ncRNA showing a significant (*p* < 0.05) increase in H3K9ac with concurrent gene expression upregulation (Figure 1F).

**Figure 2:**
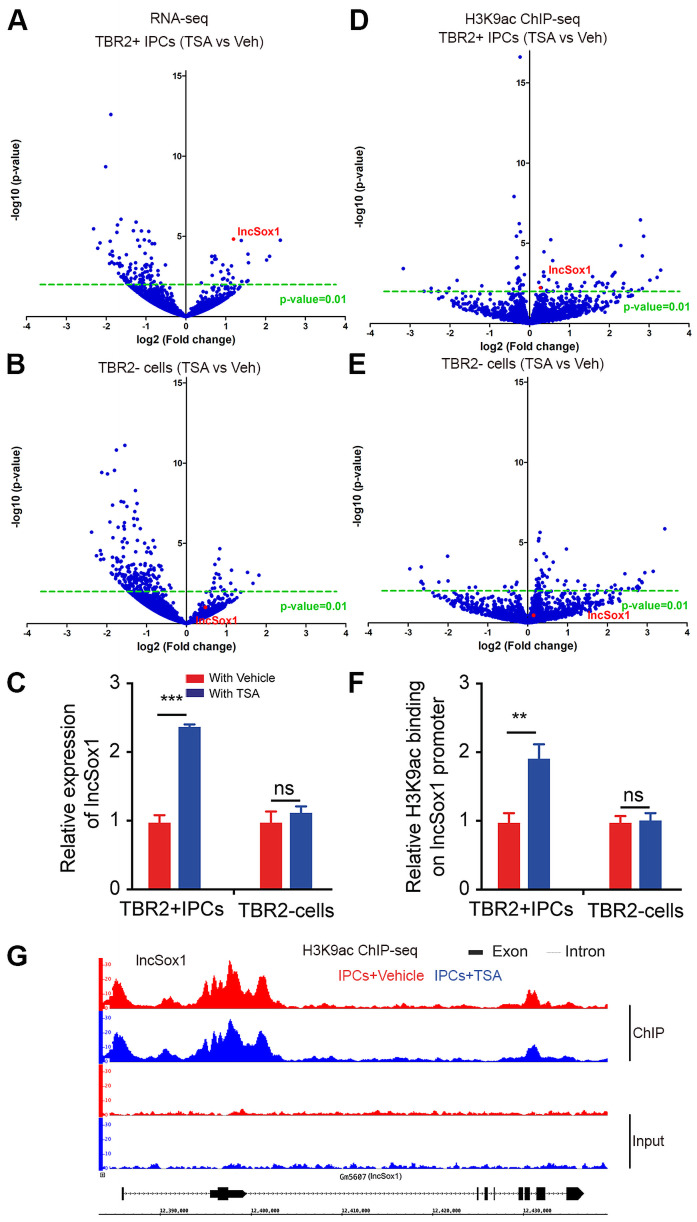
lncSox1 promoter is a target for TSA-mediated H3K9ac upregulation leading to increased lncSox1 expression in IPCs. (**A**–**F**) Volcano plots showing upregulated and downregulated genes (*p* < 0.01) in the TSA-treated IPC (**A**,**D**) and non-IPC (**B**,**E**) transcriptome compared with their vehicle-treated counterparts. lncSox1 has been highlighted in the expression profile as significantly upregulated in (**A**,**D**) but with low expression in (**B**,**E**). (**C**,**F**) Graphs showing relatively increased expression of lncSox1 (**C**) and H3K9ac binding (**F**) in TBR2+ cells following TSA treatment compared with control, whereas lncSox1 expression (**C**) and H3K9ac binding (**F**) remained unchanged in TBR2− cells upon TSA treatment. (**G**) Genome browser view of the level and distribution of H3K9ac along the gene body of IncSox1 in TSA-treated (blue) and vehicle-treated IPCs (red). Input (lower two rows) and distributions after immunoprecipitation (upper two rows) are indicated. Values are presented as means ± SEMs. Asterisk (*) denotes level of statistical significance (** *p* < 0.001, *** *p* < 0.0001), and ns (not significant) denotes no statistical difference.

As presented in Figure 1F, *lncSox1* expression seemed to be particularly upregulated in the sorted IPCs treated with TSA. We further analyzed if there was any element of selectivity in targeting *IncSox1* among the pool of ncRNAs with increased expression in TBR2+ IPCs in response to the H3K9ac enhancement. Indeed, we found *lncSox1* as a top upregulated gene in the transcriptome of the TBR2+ IPCs treated with TSA (Figure 2A). However, while the expression of ncRNAs was upregulated in TBR2+ cells as a result of TSA treatment, the expression of *lncSox1* was not significantly (*p* < 0.01) increased in non-IPCs in the TSA-treated brain (Figure 1G and Figure 2B).

Further expression analysis showed that under the condition of TSA treatment there was a more than 2-fold increase in *IncSox1* expression in TBR2+ IPCs compared with TBR2− cells (Figure 2C). The observation is consistent with our previous report in which we showed that a small set of genes are specifically upregulated in IPCs due to TSA treatment [6]. A similar trend in expression discrepancy was observed for H3K9ac level on *IncSox1* promoter following H3K9ac ChIP-seq in TBR2+ IPCs compared with TBR2− cells (Figure 2D vs. Figure 2E).

Accordingly, we found a difference in promoter binding of H3K9ac in TBR2+ IPCs as compared with TBR2− cells, showing almost a 2-fold upregulation in the latter (Figure 2F). The gene body of *IncSox1* displayed a high level of H3K9ac, particular in the promoter region (Figure 2G). Together, our observation here gives an impression of an upregulated gene expression inductive effect of increased H3 acetylation at the promoter of *IncSox1* in TBR2-expressing IPCs following HDAC inhibition by TSA treatment.

### 3.3. Mouse IPCs Have Low Expression and Promoter H3K9ac Level of lncSox1 Compared with Human IPCs

**Figure 3:**
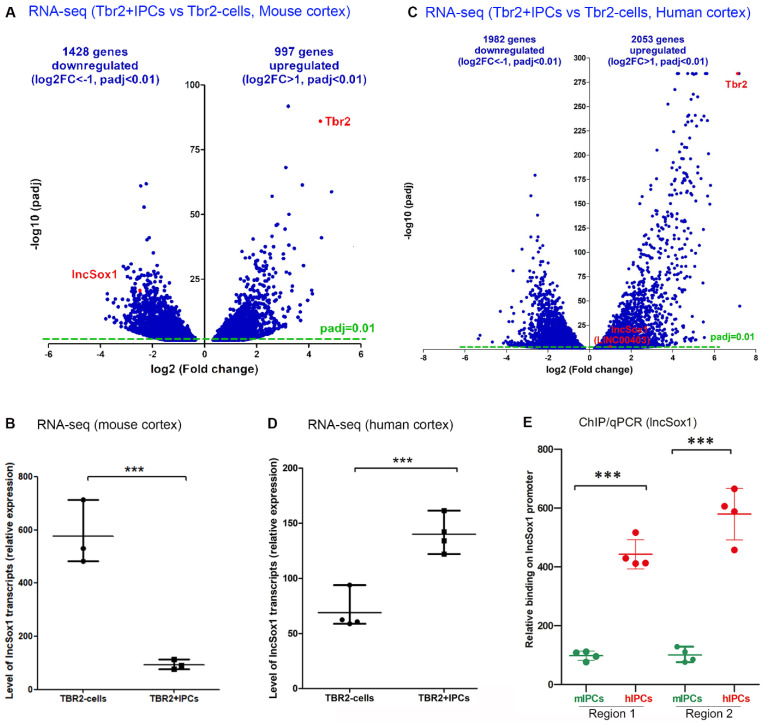
Low baseline level of H3K9ac at lncSox1 promoter and lncSox1 expression in IPCs. (**A**) Volcano plot showing upregulated and downregulated genes (*p* < 0.01; FC > 1.0) in the developing mouse cortex transcriptome. The gene expression profile shows two clusters of genes: IPC and non-IPC genes, with *lncSox1* highlighted as one of the upregulated non-IPC genes. (**B**) Graph showing relative expression of *lncSox1* in TBR2− cells compared with TBR2+ cells in mouse cortex. (**C**) Volcano plot showing upregulated and downregulated genes (*p* < 0.01; FC > 1.0) in the developing human cortex transcriptome. The gene expression profile shows two clusters of genes: IPC and non-IPC genes, with *lncSox1* highlighted as one of the upregulated IPC genes. (**D**) Graph showing relative expression of *lncSox1* in TBR2− cells compared with TBR2+ cells in human cortex. (**E**) Graph showing relative levels of H3K9ac at *lncSox1* promoter in IPCs obtained from two different developing cortical regions in mouse and human. Values are presented as means ± SEMs. Asterisks (***) denote level of statistical significance (*** *p* < 0.0001). Abbreviations: mIPCs, mouse intermediate progenitor cells; hIPCs, human intermediate progenitor cells.

We found in RNA-seq data that the expression of *lncSox1* in TBR2-expressing cells in the developing mouse cortex (Figure 3A,B) is lower than that in human (Figure 3C,D). Among 1428 genes normally downregulated in TBR2+ cells, *lncSox1* ranks as one of the top genes with decreased expression (*p* < 0.01, |FC > 1.0|) in the mouse cortex (Figure 3A). However, TBR2+ cells in the human cortex displayed a high level of *lncSox1* expression, hence it was found among the 2053 genes upregulated (*p* < 0.01, |FC > 1.0|) in the human TBR2-expressing cells (Figure 3C,D).

Interestingly, the relative amount of *lncSox1* transcripts in TBR2− cells is much higher than that found in TBR2+ IPCs in mouse (Figure 3B), whereas the converse is true for that of human (Figure 3D). Indeed, expression analysis in the developing (E14.5) mouse cortex shows *lncSox1* expression is concentrated in the ventricular zone (VZ) of the neocortex, where majority of cells are radial glial cells (RGCs) (Figure S2A). Along this line, scRNA-seq analysis [17] revealed that *lncSox1* is expressed in non-IPCs in mouse cortices with its highest level seen in RGCs and early-born neurons (EN) (Figure S2B). On the other hand, IPCs and late-born neurons (LN) are very low in *lncSox1* expression (Figure S2B).

Of note, the low expression of *lncSox1* correlates with a low promoter H3K9ac signal at the *lncSox1* locus in the TBR2+ IPCs found in the mouse cortex (Figure S2C). ChIP and qPCR experiments comparing the level of H3K9ac in TBR2+ IPCs indicated a significantly lower level of *lncSox1* promoter H3K9ac in mouse IPCs than in human IPCs (Figure 3E and Figure S2C).

The finding indicates that the normal differential expression of *lncSox1* in TBR2+ IPCs in mouse and human is linked to a difference in H3K9ac enrichment at the *lncSox1* promoter region, which is high in human IPCs and likely responsible for the *lncSox1* upregulation therein. Thus, this observation is a plausible explanation for why there is increased *lncSox1* expression in TBR2+ IPCs following induction of high H3K9ac as a result of TSA treatment (Figure 1F and Figure 2A–F).

### 3.4. Targeted H3K9 Acetylation Increases Promoter H3K9ac and Expression of lncSox1

**Figure 4:**
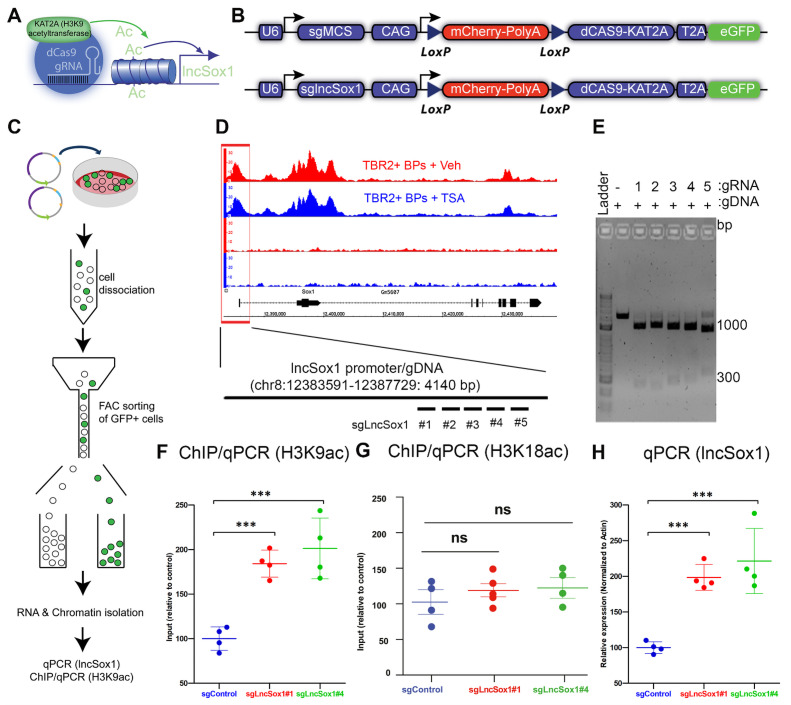
Editing H3K9 acetylation specifically at lncSox1 promoter has implications for enhanced lncSox1 expression. (**A**) Illustrative overview of the CRISPR/dCas9-mediated installation of H3K9ac at the *lncSox1* promoter to augment lncSox1 expression. (**B**) Schema showing CRISPR/dCas9 constructs used for validating the phenotypic effect of H3 acetylation at the *lncSox1* promoter. (**C**) Diagrammatic representation of the Neuro2A cell culture system used for testing the CRISPR/dCas9 constructs for editing H3K9ac at *lncSox1* promoter followed by FAC sorting of transfected (GFP+), and isolation of chromatin and RNA for the quantification of H3K9ac and *IncSox1* expression, respectively. Green and white circles represent GFP-expressing and non-GFP-expressing cells, respectively. (**D**) Genome browser view showing the level and distribution of H3K9ac along the gene body of *IncSox1* in TSA-treated IPC (blue) and vehicle-treated IPCs (red). Input (lower two rows) and distributions after immunoprecipitation (upper two rows) are indicated. The various tested gRNAs targeting different regions of IncSox1 promoter for H3K9ac editing are indicated. (**E**) Image of agarose gel showing the cutting efficiency of each gRNA-Cas9 complex tested on a 4140 bp-long PCR product of IncSox1 promoter region. (**F**–**H**) Graphs showing quantitative increase in the level of H3K9ac at *IncSox1* promoter (**F**), no measurable change in the level of H3K18ac used as negative control (**G**), and elevation in the expression of *IncSox1* (**G**) in the cultured Neuro2A cells transfected with the gLncSox1 #1 and #4. Values are presented as means ± SEMs. Asterisks (***) denotes level of statistical significance (*** *p* < 0.0001), and ns (not significant) denotes no statistical difference.

To validate that increased H3K9 acetylation specifically at the *lncSox1* locus is necessary for promoting the expression of *lncSox1*, we employed a CRISPR/dCas9-based system [6,18] which allowed targeted deposition of H3K9ac marks at the *lncSox1* locus (Figure 4A).

We generated plasmid constructs (gLncSox1-dCas9-KAT2A-T2A-eGFP) bearing DNA sequences that encoded a guide RNA (gRNA), and dCas9 (a modified Cas9 without nuclease function) fused to the acetyltransferase KAT2A, and GFP as a fluorescent reporter (Figure 4B). Thus, the approach constitutes targeted epigenome editing leading to the remodeling of the H3K9ac landscape at the regulatory (promoter) region of *lncSox1*.

To target H3K9 acetylation at *lncSox1* promoter in a cell type-specific manner, we employed the Cre/loxP system in our epigenome editing construct, in which an mCherry-polyA sequence flanked by loxP sites was placed in front of the dCas9-KAT2A sequence (Figure 4B). Using sorted cultured neurons (Neuro2A), we validated several designed gRNAs capable of targeting the *lncSox1* promoter. Multiple *lncSox1* sgRNAs (sglncSox1 #1–5), were used to target the *lncSox1* promoter for acetylation by the H3K9ac writer KAT2A (Figure 4C–E).

Upon sorting the Neuro2A cells co-transfected with the H3K9ac-installing construct together with pCAG-Cre plasmid (i.e., GFP-expressing cells), and performing ChIP-qPCR experiment (Figure 4C), we observed a significant (*p* < 0.0001) elevation in the level of H3K9ac at the *lncSox1* promoter due to deposition of H3K9ac under the gLncSox1#1 and gLncSox1#2 as compared with the control construct (gControl) (Figure 4F).

In addition to assessing H3K9 acetylation, we examined H3K18 acetylation as a control. As expected, targeting the *lncSox1* promoter with our CRISPR-based epigenome editing system selectively increased H3K9 acetylation without affecting H3K18 acetylation levels at the same promoter region (Figure 4G). These findings support the specificity of the epigenome editing approach and indicate that the observed transcriptional effects are associated with targeted changes in H3K9 acetylation rather than a general increase in histone acetylation.

Based on qPCR analysis, we found an increased level of *lncSox1* in the isolated cells transfected with the H3K9ac editing constructs generated (Figure 4H). These results indicate validation of the CRISPR/Cas9-mediated epigenome editing system as capable of H3K9ac deposition at the promoter of *lncSox1*, which resulted in increased expression of *lncSox1* in neural cells.

### 3.5. Targeted H3K9 Acetylation at lncSox1 Promoter in IPCs Elicits Observable Cortical Phenotype

**Figure 5:**
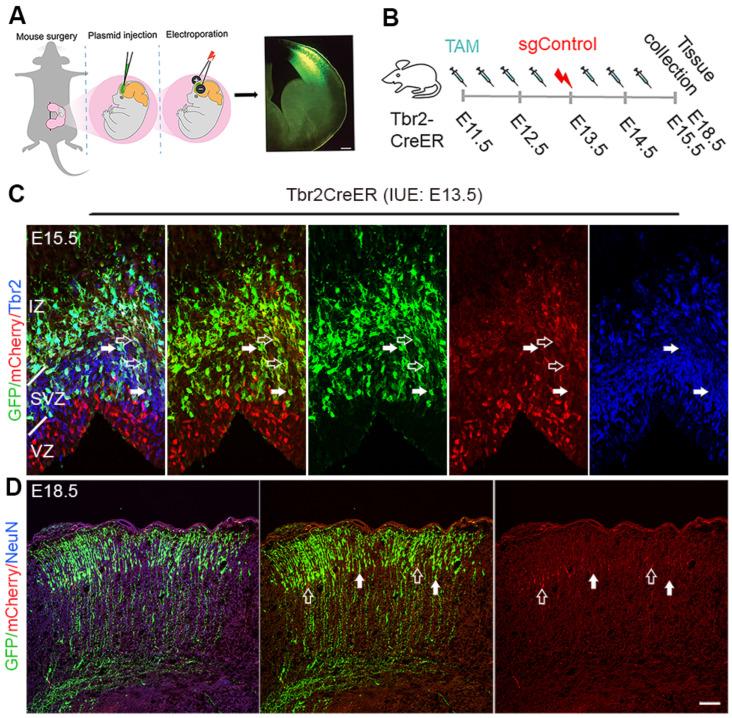
Cortical phenotype analysis following editing of H3K9ac at the promoter region of *IncSox1*. (**A**) Schematic overview of in utero electroporation of the embryonic mouse cortex with sgLncSox1 and Tbr2CreER plasmids. (**B**) Illustration of treatment of mouse embryos with tamoxifen (TAM) and Tbr2CreER via scheduled intraperitoneal injections. (**C**,**D**) Immunohistochemistry micrographs of the E15.5 (**C**) and E18.5 cortex stained for the indicated markers following the in utero electroporation-mediated H3K9ac editing at the promoter region of *IncSox1*. White arrows in (**C**,**D**) point to mCherry-non-labelled cortical cells exposed to CreER and with faint or no TBR2 labelling (**C**) in the E15.5 cortical germinal zone (**C**) and E18.5 cortical plate (**D**). Empty arrows in (**C**,**D**) point to mCherry-labelled cells with no or faint TBR2 expressing in (**C**) in the E15.5 cortical germinal zone (**C**) and E18.5 cortical plate (**D**). Abbreviations: VZ, ventricular zone; SVZ, subventricular zone; IZ, intermediate zone. Scale bars = 50 µm.

To achieve in vivo regulation of H3K9ac levels at lncSox1 promoter in IPCs, we delivered the epigenome editing constructs into tamoxifen (TAM)-treated TBR2/Eomes-CreER mouse embryos [7] via IUE at E13.5 (Figure 5A,B). Cre recombinase is driven by the IPC-specific TBR2 regulatory elements, which switch expression from mCherry to dCAS9-KAT2A and eGFP specifically in IPCs and their progenies (Figure 5A,B). To validate our cell type-specific epigenome editing system, we examined the location of mCherry+, and eGFP+ cells and their co-expression with the IPC marker TBR2. At E15.5, the majority of cells expressing high level of mCherry were found in TBR2− cells in the VZ (Figure 5C,D). As expected, most of the eGFP+ cells were found in TBR2+ cells in SVZ, and possibly nascent neuronal progenies of IPCs in IZ/CP (Figure 5C, filled arrows). At E15.5, we noted that many eGFP+ cells in SVZ/IZ also expressed mCherry at a low level, possibly due to mCherry protein stability over a few days (Figure 5C, empty arrow).

Of note, all eGFP and mCherry are not co-expressed in the cortex at a later stage, i.e., E18.5 (Figure 5D, arrows). Thus, the established cell type-specific epigenome editing system allowed addition of the epigenetic mark H3K9ac at the *lncSox1* promoter specifically in IPCs and their progenies in developing mouse cortex. This made it possible to discern the phenotype which accompanied *lncSox1* upregulation in the developing cortex due to the H3K9ac treatment or editing.

### 3.6. IPC-Specific H3K9 Acetylation Editing at lncSox1 Promoter Increases IPC Proliferation and Indirect Neurogenesis

To determine the impact of increasing the expression of *lncSox1* in IPCs through targeted increase in the promoter level of H3K9ac, we immunohistochemically analyzed the developing mouse cortex treated with our epigenome editing tool. At E14.5, the cortex electroporated with gLncSox1 plasmid displayed a greater ratio of TBR2+/GFP+ cells in the SVZ per mCherry+/TBR2− cells in the VZ than that in the control plasmid-injected cortex (Figure S3A), suggesting TBR2+ IPC population was expanded upon gLncSox1 expression.

**Figure 6:**
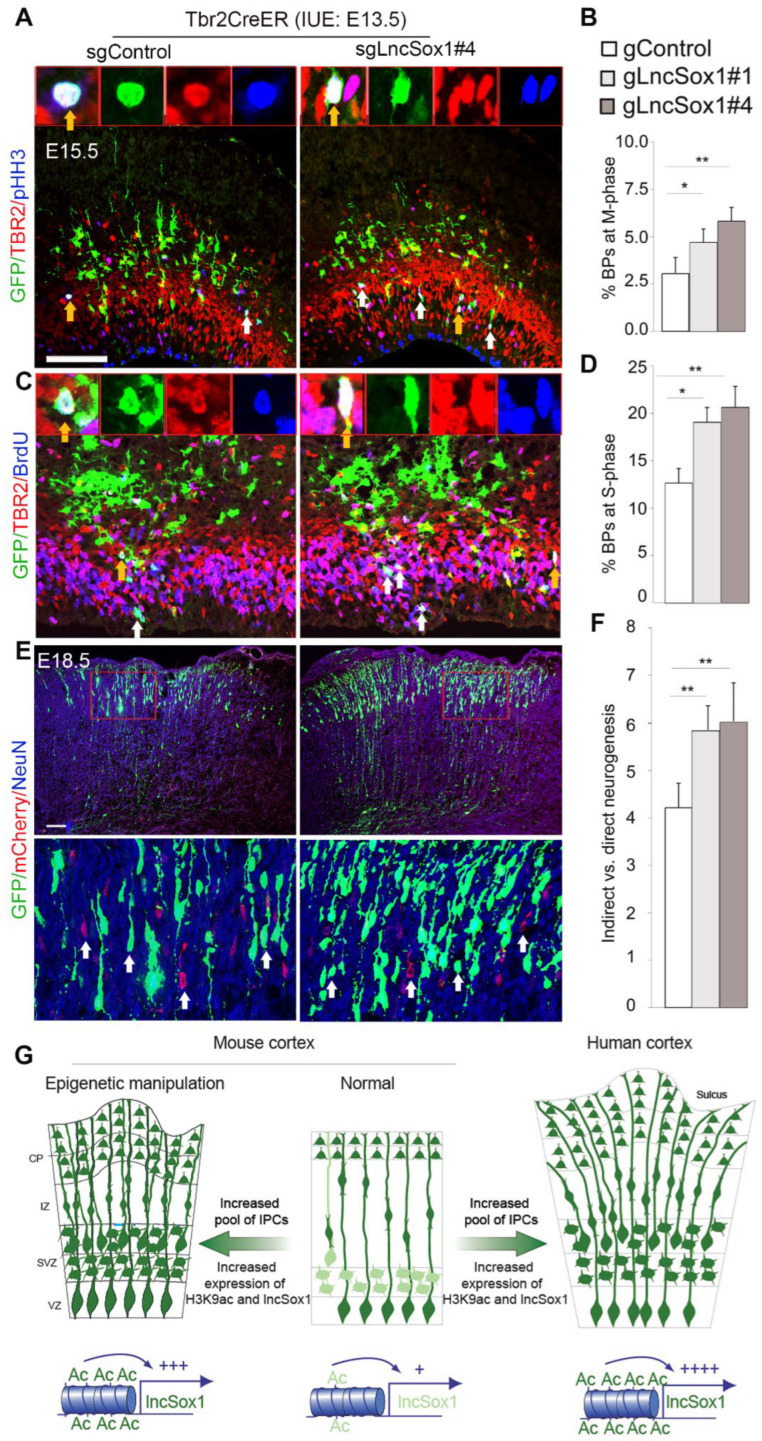
Targeted H3K9 acetylation at lncSox1 promoter encourages IPC proliferation and neurogenesis. (**A**) Micrographs showing immunostaining analysis of pHH3, TBR2, GFP in the E15.5 mouse cortex electroporated at E13.5 with sgLncSox1 and sgControl plasmids. Inserts are high magnifications of cells stained with the indicated markers and display M-phase status. Yellow arrows point to TBR2+ cells with mitotic figures and white arrows point to those without mitotic figures. (**B**) Statistical quantification of proliferating TBR2-expressing cells in M-phase in the sgLncSox1-, and sgControl-treated E15.5 cortex. (**C**) Micrographs showing immunostaining analysis of BrdU, TBR2, GFP in the E15.5 mouse cortex electroporated with sgLncSox1 and sgControl plasmids. Inserts are high magnifications of cells stained with the indicated markers and display S-phase status. Yellow arrows point to TBR2+ cells with active DNA replication and white arrows point to those without active DNA replication. (**D**) Statistical quantification of proliferating TBR2-expressing cells in S-phase in the sgLncSox1-, and sgControl-treated E15.5 cortex. (**E**) Micrographs at low (upper panels) and high (lower panels) magnifications showing GFP, mCherry, and NeuN immunostaining in the E18.5 mouse cortex electroporated at E13.5 with sgLncSox1 and sgControl plasmids. Red rectangle demarcates zoomed area of the cortical plate shown in the lower panel micrographs for quantitative analysis. White arrows point to mCherry/NeuN cells. Their lack of GFP expression indicates they are derived from non-TBR2-expressing cells or direct neurogenesis. Cells with GFP/mCherry/NeuN staining are derived from TBR2+ cells via indirect neurogenesis. (**F**) Statistical quantification of the proportion of cells undergoing direct or indirect neurogenesis in the sgLncSox1-, and sgControl-treated E18.5 cortex. Note that sgLncSox1#1 yielded a mild phenotype compared to sgLncSox1#4 (**B**,**D**,**F**). (**G**) Schema illustrating how *lncSox1* expression in the normal mouse cortex is augmented by its promoter region H3K9ac enrichment leading to intermediate progenitor pool expansion, which has implication for expansion and folding of the epigenetically manipulated mouse cortex. The epigenetic manipulation can cause the normally smooth mouse brain to gyrencephalate. Plus (+) denotes extent of gene expression activation. Values are presented as mean ± SEM. Asterisk (*) denotes level of statistical significance (* *p* < 0.05, ** *p* < 0.001), and ns (not significant) denotes no statistical difference. Scale bars = 50 µm. Abbreviations: Ac, H3K9 acetylation mark; IPCs, intermediate progenitor; VZ, ventricular zone; SVZ, subventricular zone; IZ, intermediate zone; CP, cortical plate.

We also analyzed the proliferative capacity of TBR2+ IPCs in the developing cortex (E15.5) following the augmentation of *lncSox1* expression (Figure 6A–D). An assessment of the proportion of TBR2+/BrdU+ (IPCs in S-phase) and TBR2+/pHH3+ (IPCs in M-phase) cells among the GFP-labelled TBR2+ IPCs in gControl- and gLncSox1 construct-injected cortices enabled us to determine if the H3K9ac level alterations at the *lncSox1* promoter contributed to the proliferation of IPCs.

During corticogenesis, RGCs are actively involved in direct neurogenesis and undergo indirect neurogenesis via IPCs. To exclude the possibility that our IPC-specific H3K9ac editing system does not influence direct neurogenesis, we also used the same approach to deliver the expression plasmids in the E13.5 cortex. Given that RGCs undergo only one division in 24 h at mid-gestation [19], we collected tissue at E14.5 and performed double immunostaining for mCherry and neuronal markers Tuj or HuCD to quantify the neuronal production directly from mCherry+ RGCs. In the control, electroporated (mCherry+) cells were located in the SVZ/IZ and some co-expressed Tuj1/HuCD (22% ± 2.6% of all mCherry+ cells), representing differentiated neurons generated directly from RGCs (Figure S3B–E). Compared to control, the gLncSox1 plasmid-electroporated cortex had a similar proportion of mCherry+/Tuj1/HuCD+ neurons among the targeted mCherry+ cells, indicating that the IPC-specific gLncSox1 activation did not affect direct neurogenesis.

Remarkably, the increase in promoter H3K9ac level of *lncSox1* by our epigenome editing tool increased the amount (percentage) of basal mitosis (BrdU+ or TBR2+/pHH3/GFP+) among targeted IPCs (TBR2+/GFP+; Figure 6A–D).

To examine whether the IPC-specific gLncSox1 overexpression promotes indirect neurogenesis, we quantified the number of GFP+/NeuN+ or Satb2+ neurons in the gLncSox1, or gControl plasmids-electroporated cortices at E18.5. Because direct neurogenesis is not different between the gLncSox1 and empty vector plasmid (EV)-electroporated cortices, we used mCherry+/NeuN+ or mCherry+/Satb2+ neurons as internal control for comparison (Figure 6E). Our comparative analysis indicated that the gLncSox1-electroporated cortices have a greater ratio of GFP+ neurons per mCherry+ neurons than that of EV plasmids-electroporated cortices and indicative of increased indirect neurogenesis (Figure 6F).

Because we found that apoptosis in the cortex, and direct neurogenesis were not significantly different between gLncSox1 gain-of-function effect and that of control, these findings indicate that IPC-specific activation of gLncSox1 promotes cortical neurogenesis via augmented IPC proliferation.

## 4. Discussion

Brain complexification can be partly described on the basis of the adoption and/or operationalization of new and advanced regulatory mechanisms or the hierarchical prominence of such mechanisms in the course of evolution. Proliferative neurogenic cells such as TBR2-expressing IPCs are critical determinants of brain development, and there is interspecies variation in the abundance of TBR2+ IPCs in the developing brain. Therefore, an understanding of the molecular factors driving this variation would afford an *evo–devo* insight into brain organogenesis and expansion. For instance, emergence of the evidence that H3 acetylation in basal progenitor cells is a key mechanism underlying cortical development [6] has provoked questions about how the neuroepigenome contributes to the evolutionary expansion of the mammalian brain. However, it is challenging to target specific epigenetic events or factors to study their role in specific populations of cells. We attempted answering such questions by seeking to unravel the precise regulatory epigenetic mechanisms which may drive the proliferative capacity of IPCs in the developing cortex, with speculative implication for brain evolution. Our quest brought into focus the exploration of targeted gene regulation in a purified population of neural cells. This led to the concept of combining the epigenome editing strategy and FACS-mediated cell isolation protocol to cell type-specifically probe for the epigenetic factor(s) involved in IPC biogenesis during cortical expansion. The advantage offered by epigenome editing is the effective manipulation of the genomic phenotype without direct interference of the nucleotide sequence in the gene(s) of interest.

We went about our neuroepigenome editing objective by initially treating the developing mouse cortex with TSA to chemically inhibit histone deacetylation, which is essentially an indirect approach to upregulate histone acetylation [6]. We then isolated IPCs from the TSA-treated brains. The use of the intranuclear immunostaining FACS method for purifying IPCs was to ensure comprehensive isolation of TBR2-expressing IPCs in the developing cortex [11]. Next, we examined the IPC transcriptome for key changes in response to the histone acetylation alteration. The RNA-seq analysis and Chip-seq experimentation were used to probe for epigenetic changes which distinguish TBR2+ cells from TBR2− cells in the context of response to increase in histone acetylation. Our observation of overt changes in ncRNA expression in the sorted TSA-treated TBR2+ IPCs led to the identification of *lncSox1* expression as a major downstream effect of histone acetylation upregulation in IPCs. This outcome is consistent with the observation that targeted increase in histone acetylation at promoters and enhancers is sufficient to enhance gene expression [20]. Indeed, by using our epigenome editing tool, we were able to efficiently deposit H3K9ac at the promoter of *lncSox1* which resulted in its increased expression. The strategy of targeting different promoter regions of *lncSox1* gene with multiple *lncSox1* gRNAs was to ensure efficient targeting leading to adequate achievement of the desired manipulative effect, i.e., H3K9ac addition at the promoter region. This strategy has gained popularity because it helps in achieving robust activation of genes [20,21,22,23,24,25,26,27].

Application of the CRISPR-dCas9 platform to effect durable gene expression regulation in the neuroepigenome is fast becoming one of the most reliable and highly efficient strategies for identifying the specific contribution of epigenetic factors, including chromatin marks, in neurodevelopment [28]. A fascinating feature of CRISPR-dCas9-mediated epigenome editing is the programmability of the gRNA component, which allows targeted manipulation at specific gene loci [29]. The choice of *lncSox1* as the target gene for epigenome editing was informed by the outcomes of our RNA-seq and ChIP-seq experiment as commonly done to validate epigenome editing protocols (reviewed in [28]). Our gLncSox1 was able to efficiently target the acetyltransferase KAT2A to the promoter region of *lncSox1* gene for H3K9ac installation leading to the inductive upregulation of *lncSox1* expression. The use of TBR2-Cre for the activation of the CRISPR-dCas9 construct ensured the cell type-specific targeting of TBR2-expressing IPCs in vivo. Other previously reported ways of increasing the efficiency of an epigenome editing machinery worth considering for improving CRISPR-dCas9-dependent epigenome engineering specificity include (i) using shorter (18–19 base pairs) gRNAs to increase binding complementarity [30], (ii) point mutating a positively charged domain in the CRISPR-Cas9 structure to reduce gRNA off-targeting [31], and (iii) modifying the secondary structure of gRNAs by adding a hairpin loop to inhibit Cas9 off-target binding [32].

To validate our epigenome editing scheme, we sought to analyze the outcome of editing H3K9ac at the promoter region of *lncSox1* in IPCs during cortical development. The observed striking consequence of increase in the IPC pool can be reasonably linked to the upregulation of *lncSox1* following the H3K9ac enhancement manipulation. Our finding that the lncSox1-enriched IPCs exhibit enhanced proliferative capacity explains the demonstrable increase in the population of the IPCs and the resultant increase in neurogenesis. This observation connects well with previous reports that *lncSox1 (Sox1ot)* expression is upregulated in differentiated neural stem cells in a temporal manner [33]. By interacting with Sox1, a proneural factor, lncSox1 may be involved in several independent pathways necessary for neurogenesis [33,34] and also in regulating cortical expansion driven by dynamic H3K9ac levels at the *lncSox1* promoter region (this study and [6,35]). Thus, the effect of our targeted epigenome editing experiment in the developing mouse cortex offers an opportunity to uncover novel mechanisms in specific populations of cells which can elucidate developmental and pathophysiological processes in the brain. Indeed, the emerging role of lncRNAs, including *lncSox1*, in brain development [36,37] points to the possible implication of *lncSox1* in neurodevelopmental and neuropsychiatric disorders when misexpressed [38].

The epigenome editing paradigm employed in this study can be fraught with challenges considered as classic limitations of epigenome editing platforms [39,40]. Notable among them are efficiency issues stemming from non-specific editing, and interference from endogenous mechanisms which can destabilize or reverse modifications. It is worth adding that there can be variations in the modifiability of genes, which can have implications for differences in the durability of modifications and outcomes. There may be an element of context in selecting or targeting cells for epigenome editing. In our case, TBR2 may be transiently and differentially expressed in proliferating and differentiating IPCs. This implies that the interpretation and generalization of cellular effects would need to be based on additional layers of cell identity.

## 5. Conclusions

Put together, histone acetylation has been found to drive lncRNA-mediated regulation of cortical neurogenesis. By means of our epigenome editing paradigm, the histone mark H3K9ac has been identified as an epigenetic signature of IPCs notably mediated by selected ncRNAs such as *lncSox1* (lncRNA *Sox1ot*). We speculate that the uncovered epigenetic H3K9ac−lncSox1 regulatory axis in corticogenesis may have been evoked as an evolutionary requirement for increasing the neural progenitor pool in the developing mammalian gyrencephalic cortex (Figure 6G).

The experimental model in this study can be used for targeted gene editing in a specific population of cells, thus making it possible to determine the contribution of (un)known (epi)genetic factors in pertinent cell biological processes. Such targeted molecular probing can lend cues for therapeutic strategies for remedying disorders attributed to abnormalities in cell niches, and with the potential of achieving high fidelity treatment outcomes [39,40].

## Data Availability

RNA-seq and ChIP-seq data have been deposited at the GEO under the accession number GSE168298 and are publicly available as of the date of publication.

## Supplementary Materials

The following supporting information can be downloaded at: https://www.mdpi.com/article/10.3390/biology15141110/s1, Figure S1: Expressions of ncRNAs within and outside the ventricular zone of the developing mouse cortex; Figure S2: Expression of lncSox1 in mouse cortex is mainly found in non-IPCs and associated with low promoter levels of H3K9ac; Figure S3: H3K9ac-mediated increase in *lncSox1* expression promotes generation of IPCs without altering early-stage neurogenesis; Table S1: Differential binding of H3K9ac at ncRNA promoters between TBR2+ IPCs in TSA- and vehicle-treated embryonic cortex (as a Supplemental Spreadsheet); Table S2: Differential ncRNA gene expression between TBR2+ IPCs in TSA- and vehicle-treated embryonic cortex (as a Supplemental Spreadsheet); Table S3: Differential binding of H3K9ac at ncRNA promoters between TBR2− cells in TSA- and vehicle-treated embryonic cortex (as a Supplemental Spreadsheet); Table S4: Differential ncRNA gene expression between TBR2− cells in TSA- and vehicle-treated embryonic cortex (as a Supplemental Spreadsheet); Table S5: Statistical analyses (as a Supplemental Spreadsheet); Table S6: List of primers for qPCR and ChIP-qPCR (as a Supplemental Spreadsheet).

## Abbreviations

The following abbreviations are used in this manuscript:

*lncSox1*	Long non-coding Sox1
H3K9ac	Acetylation of histone H3 at 9th lysine residue
*Sox1ot*	Sox1 overlapping transcript
lncRNA	Long non-coding Ribonucleic acid
CRISPR	Clustered regularly interspaced short palindromic repeats
dCas9	Dead CRISPR-associated protein 9
IPC	Intermediate progenitor cell
TBR2	T-box brain protein 2
TSA	Trichostatin A
IUE	In utero electroporation
HDACi	Histone deacetylase inhibitor
ChIP-Seq	Chromatin immunoprecipitation sequencing
FACS	Fluorescence-activated cell sorting
IHC	Immunohistochemistry
qRT-PCR	Quantitative reverse transcription polymerase chain reaction
sgRNA	Single guide ribonucleic acid
qPCR	Quantitative polymerase chain reaction
Veh	Vehicle
EV	Empty vector
ncRNA	Non-coding Ribonucleic acid
mIPCs	Mouse intermediate progenitor cells
hIPCs	Human intermediate progenitor cells
VZ	Ventricular zone
SVZ	Subventricular zone
IZ	Intermediate zone
RGCs	Radial glial cells
EN	Early-born neurons
LN	Late-born neurons
scRNA-seq	Single cell ribonucleic acid sequencing
gRNA	Guide ribonucleic acid
TAM	Tamoxifen
KAT2A	Lysine acetyltransferase 2A
eGFP	Enhanced green fluorescent protein
CreER	Inducible cre-loxP recombination
DAPI	4′,6-diamidino-2-phenylindole
