# Supplementary material for: Histone H3 Acetylation at Sox1ot Promoter by Targeted Epigenome Editing Augments Proliferation of Intermediate Progenitors in Developing Cortex
Source: Biology (Basel). 2026 Jul 9;15(14):1110. doi: 10.3390/biology15141110 (PMC13403965; doi:10.3390/biology15141110)
Supplement: Supplementary file 1 [file biology-15-01110-s001.zip › Supplemental information_Sokpor et al.pdf]

**SUPPLEMENTARY INFORMATION**

***Supplementary figures***

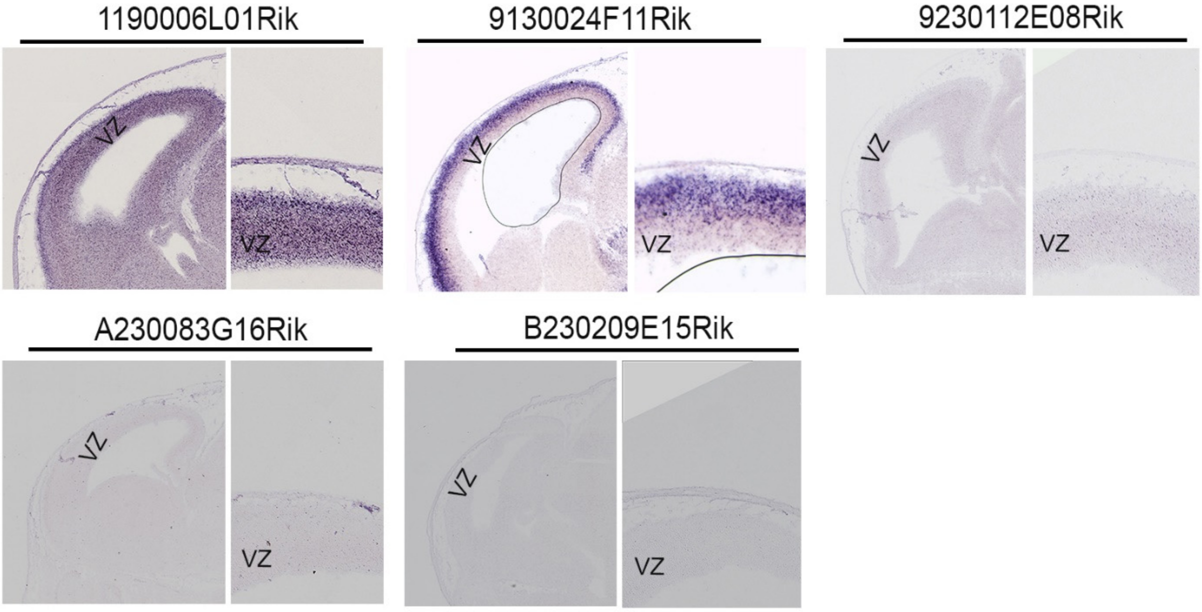


**Figure S1: Expressions of ncRNAs within and outside the ventricular zone of the developing mouse cortex.**

Micrographs obtained from GenePaint showing *in situ* hybridization of selected ncRNAs in the developing mouse cortex and indicate that whereas some ncRNAs are expressed in the ventricular zone, others are predominantly expressed in the (presumptive) cortical plate.

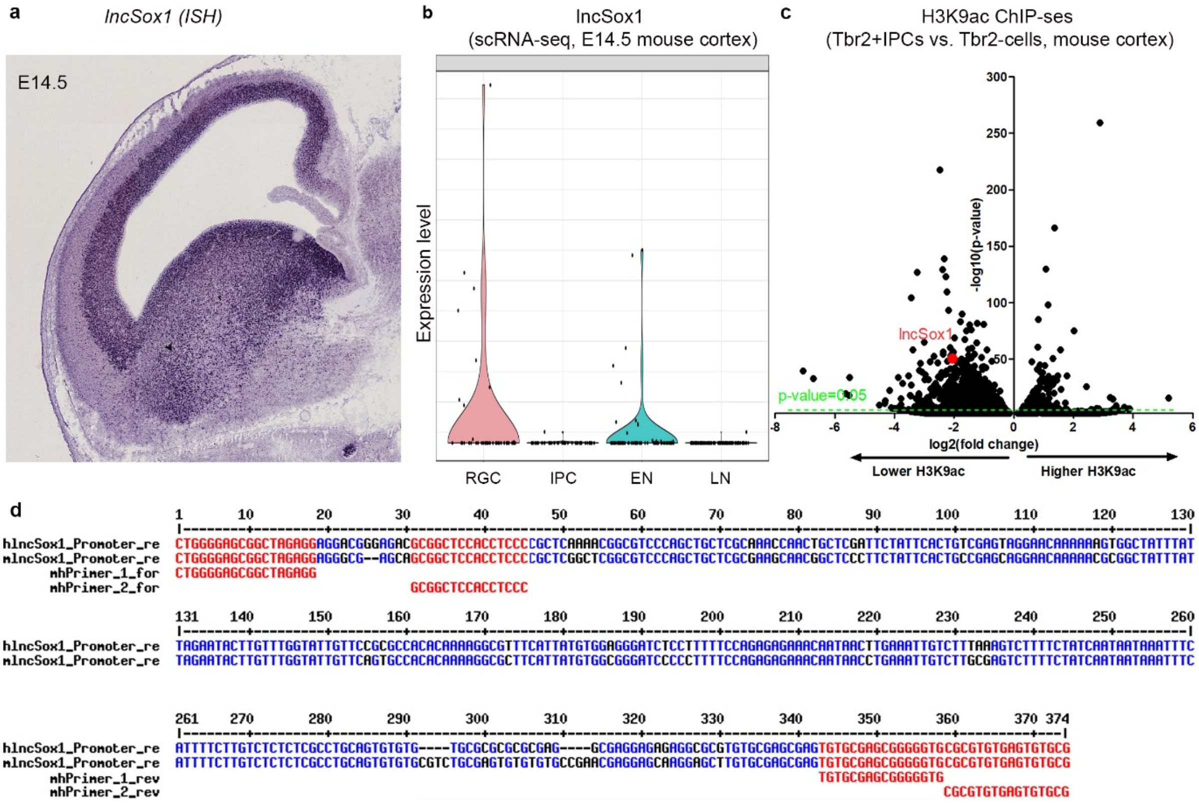


**Figure S2: Expression of lncSox1 in mouse cortex is mainly found in non-IPCs and associated with low promoter levels of H3K9ac.**

(a) Micrograph showing *in situ* hybridization (ISH) of *lncSox1* in the E14.5 mouse cortex with overt signal intensity in the ventricular zone. (b) Image showing single cell RNA sequencing analysis of *lncSox1* expression profile in the E14.5 mouse cortical cells. (c) Volcano plots showing H3K9ac distribution profile of IPC and non-IPC genes following ChIP/qPCR sequencing analysis. The *lncSox1* gene is highlighted in red. (d) DNA sequence alignment for two ortholog regions of mouse (m) and human (h) *lncSox1* promoter and primers, which were used in ChIP/qPCR experiment (see also Fig. 3E). Note that mhPrimer_1 set was used to amplify the region 1 of both mouse and human *lncSox1* promoter. The mPrimer_2 and hPrimer_2 sets, which have similar sequence, were used to amplify the region 2. Abbreviations: RGC, radial glial cell; IPC, intermediate progenitor cell; EN, early-born neuron; LN, late-born neuron.

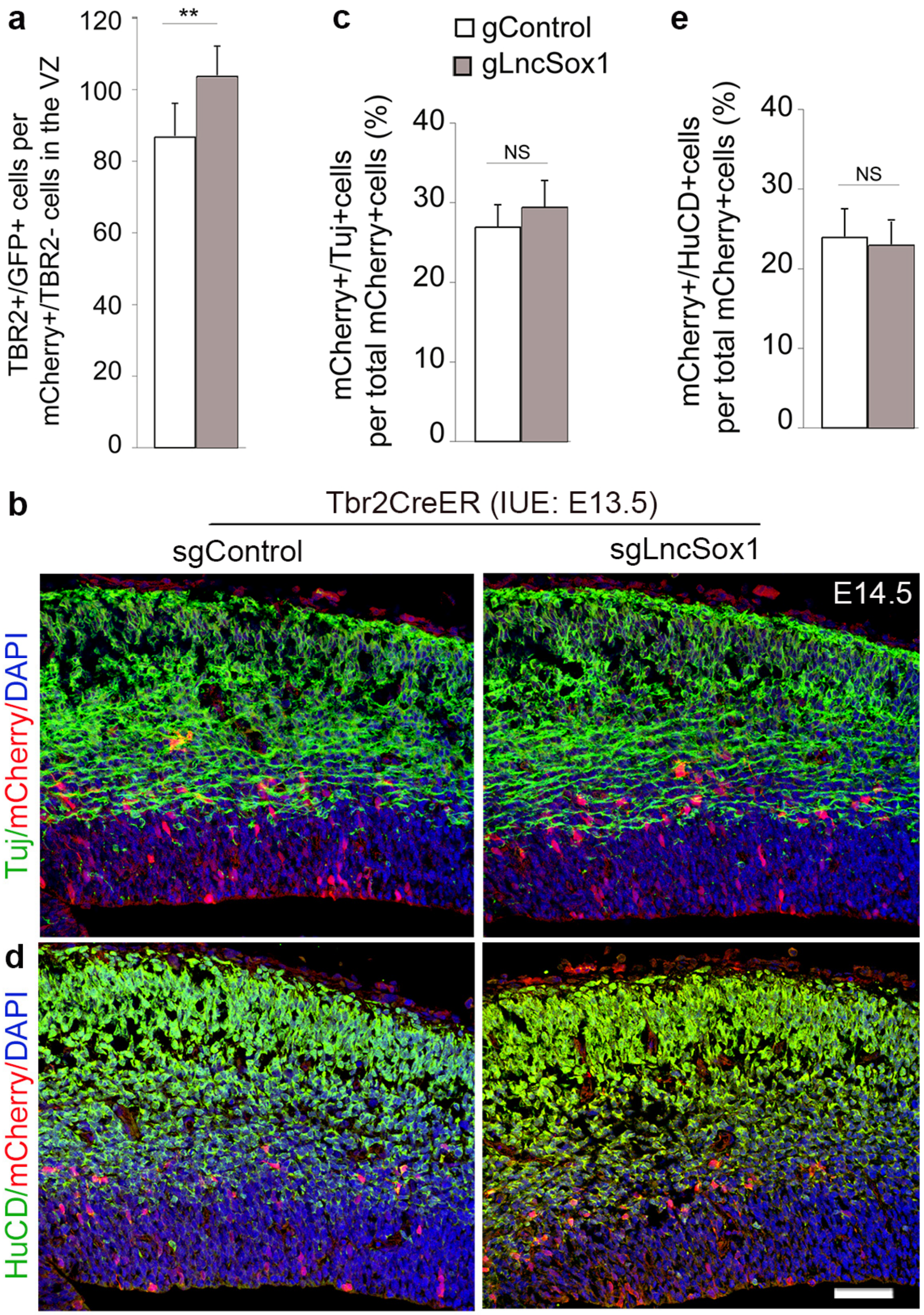


**Figure S3: H3K9ac-mediated increase in lncSox1 expression promotes generation of IPCs without altering early-stage neurogenesis.**

(a, b) Bar graphs (a) and immunomicrographs (b) showing significant increase in Tbr2 expressing cells following treatment of the E14.5 mouse cortex with sgLncSox1 compared to control. (c-e) Bar graphs (c, e) and immunomicrographs (d) showing no significant difference in the number of early-born neurons following treatment of the E14.5 mouse cortex with sgLncSox1 compared to control. Values are presented as means ± SEMs (**$p$ < 0.001, NS: not significant). Scale bar = 50 μm.

***Supplemental data files***

- **Table S1:** Differential binding of H3K9ac at ncRNA promoters between TBR2+ IPCs in TSA- and vehicle-treated embryonic cortex (as spreadsheet).
- **Table S2:** Differential ncRNA gene expression between TBR2+ IPCs in TSA- and vehicle-treated embryonic cortex (as spreadsheet).
- **Table S3:** Differential binding of H3K9ac at ncRNA promoters between TBR2- cells in TSA- and vehicle-treated embryonic cortex (as spreadsheet).
- **Table S4:** Differential ncRNA gene expression between TBR2- cells in TSA- and vehicle-treated embryonic cortex (as spreadsheet).
- **Table S5:** Statistical analyses (as spreadsheet).
- **Table S6:** List of primers for qPCR and ChIP-qPCR (as spreadsheet).
